# Productive Aging Profiles Among Vietnamese War Survivors: The Role of Early-Life War Exposure and Military Service

**DOI:** 10.1093/geroni/igaf122.815

**Published:** 2025-12-31

**Authors:** Bussarawan Teerawichitchainan, Sara Hamm, Zachary Zimmer, Minh Nguyen

**Affiliations:** National University of Singapore, Singapore, Singapore; Mount Saint Vincent University, Halifax, Nova Scotia, Canada; Mount Saint Vincent University, Halifax, Nova Scotia, Canada; Vietnam Academy of Social Sciences, Hanoi, Ha Noi, Vietnam

## Abstract

War disrupts millions of lives, particularly in low- and middle-income countries (LMICs), where armed conflicts are most frequent. While much is known about war’s detrimental effects on physical and psychological health, its long-term impact on productive aging remains unclear. This study investigates how early-life war exposure affects productive aging among older Vietnamese war survivors and examines the moderating role of past military service and war trauma exposure. Data come from the Vietnam Health and Aging Study (N = 2,447 war survivors aged 60+). Latent class analysis identifies patterns of engagement across five domains: work, in-kind support, caregiving, community involvement, and self-development. Multinomial logistic regressions assess relationships between war exposure intensity, military role, and engagement patterns. Our analyses identify five patterns of later-life productive engagement: High Engagers, Altruistic, Low Engagers, Civic Developers, and Active Workers. Greater exposure intensity is associated with profiles indicative of higher levels of engagement. Military roles moderate this relationship, with formal military veterans more likely to belong to the Active Workers class under high exposure conditions, while informal military members are more likely to be classified as Low Engagers. To promote productive aging in conflict-affected LMICs, policymakers should consider the long-term impacts of early-life war exposure and past military service. Addressing inequalities and introducing interventions earlier in the life course could enhance effectiveness.

